# Perspectives on Underlying Factors for Unhealthy Diet and Sedentary Lifestyle of Adolescents at a Kenyan Coastal Setting

**DOI:** 10.3389/fpubh.2018.00011

**Published:** 2018-02-09

**Authors:** Derrick Ssewanyana, Amina Abubakar, Anneloes van Baar, Patrick N. Mwangala, Charles R. Newton

**Affiliations:** ^1^Centre for Geographic Medicine Research Coast, Kenya Medical Research Institute (KEMRI), Kilifi, Kenya; ^2^Utrecht Centre for Child and Adolescent Studies, Utrecht University, Utrecht, Netherlands; ^3^Department of Public Health, Pwani University, Kilifi, Kenya; ^4^Department of Psychiatry, Warneford Hospital, University of Oxford, Oxford, United Kingdom

**Keywords:** adolescent behavior, sedentary lifestyle, dietary behavior, sub-Saharan Africa, qualitative research

## Abstract

Unhealthy diet and physical inactivity are among the key modifiable risk factors for non-communicable diseases, such as diabetes and cardiovascular disease. Although such diseases often only appear in adulthood, these behaviors are typically initiated or reinforced already during adolescence. However, knowledge on underlying factors for adolescents’ unhealthy dieting and physical inactivity in sub-Saharan Africa (SSA) is poor. We conducted in-depth interviews and focus group discussions to explore the perceptions of a diverse group of 78 young people of 10–19 years of age, which also included some adolescents living with HIV, as this is an emerging group in the HIV/AIDS epidemic in many parts of SSA. In addition, 10 stakeholders, such as teachers, clinicians, and staff from organizations at the Kenyan coast and seven young adult community representatives informed us on: (a) adolescents’ unhealthy food choices and their forms of sedentary behavior; (b) predisposing factors; and (c) protective factors against unhealthy food choices and sedentary behavior of adolescents living in Kilifi County. The findings reveal that adolescents occasionally access nutritious foods, such as fruits, vegetables, and animal protein. However, there is a growing tendency to consume unbalanced diets with high intake of carbohydrates, oily foods, and consumption of sugar dense processed foods and drinks. Sports and domestic chores were found to be major sources of physical activity. Sedentary lifestyles characterized by a long-time sitting and chatting, watching sports games and movies were described. Adolescents living with HIV did not indicate any divergent perceptions from those of other adolescents relating to diet and physical activity, but mentioned health-related conditions, such as medication, asthma, and low body weight, as a risk factors for sedentary lifestyle. Using a Socio-Ecological model, our findings suggest that risk factors are numerous and interrelated, especially at intrapersonal, interpersonal, and community level. The negative influences at an intrapersonal level were as follows: body image concerns, attitudes and misconceptions, substance use behavior, and taste for unhealthy foods. In the interpersonal domain, household poverty and parenting practices that condone unhealthy habits were identified risk factors. Availability of affordable unhealthy foods, high prices for nutritious food, farming practices, gambling, and influx of transportation alternatives in the community were interrelated but also had relationships with intrapersonal and interpersonal risk factors. Modernization and poor implementation of policies were discussed as enabling factors especially by stakeholders from a societal perspective. Seasonality and farming practices, school attendance, community-based services, and regulations mitigating adolescents’ engagement in gambling were identified as potential protective factors. Our findings provide a unique qualitative insight of the factors underlying adolescents’ dietary and sedentary lifestyle and highlight the need for ecological intervention approaches to address these forms of health risk behavior in a rural African setting.

## Introduction

Poor dietary behavior and insufficient physical activity rank among the key risk factors for the non-communicable disease (NCD) burden, such as diabetes and cardiovascular disease (1, 2). Although such diseases often appear in adulthood, their associated behaviors are typically initiated or reinforced already during adolescence (10–19 years) (3, 4). To this end, a global strategy on diet, physical activity, and health was endorsed by the World Health Organization in 2004, with the aim of increasing awareness, guiding policy, and action plans, monitoring underlying factors and, thus reducing the risk of NCD that stem from unhealthy diet and physical inactivity (5). It is more than a decade since this global strategy was endorsed and yet almost 81% of the global adolescent population is insufficiently physically active and policies to reduce this current state were operational in just 56% of the WHO member states as of 2013 (6). Studies have suggested a rapid global nutrition shift from consumption of traditional diet or staple foods rich in starch, plant protein, and dietary fiber toward a less healthy diet linked to NCD (7, 8). Adolescents are vulnerable as they progressively gain more control of what, when, how, and where they eat; moreover, their preferences and personal choices may gain priority over eating habits that are nurtured within the family setting (9).

In sub-Saharan Africa (SSA), which is home to 19% of the global youth population (10), the burden of NCD is projected to have the greatest increase compared to any other region by 2030 (2, 11). Already, research has pointed to an increasing trend in overweight and obesity overtime among adolescent girls and boys from SSA (12), which potentially suggests early initiation of poor dietary behavior and physical inactivity. In a systematic review, higher body weight was reported among girls compared to boys and for both groups staying in an urban setting and in households of higher socio-economic, status was linked to greater risk for overweight (12). In comparison to urban school-aged adolescents, their rural counterparts engaged in more active transportation such as walking to school and spending more time in activities of daily living such as domestic chores, although the urban dwellers engage in more organized sports activities (12). Furthermore, a report that compiled studies from the African region on risk factors among young people (age 10–24 years) stressed the current scarcity of data particularly on physical inactivity and unhealthy diet in this region, which presents limitations toward intervening with the unprecedented rise in NCD burden (2). Rapid urbanization characterized by changes in traditional transport modalities, more hours spent on socio-media, and influx of less healthy commercialized food options are linked to poor diet and physical inactivity in SSA. Other underlying factors of poor diet and physical inactivity in SSA include individual level factors, such as preferences, attitudes and beliefs, cultural practices, agricultural practices, political environment, price, availability, and accessibility (11–13). Furthermore, adolescents living with HIV are an emerging group in HIV/AIDS epidemic in many parts of SSA (14). Their HIV status adds a burden to their development, including issues of health risk taking behavior, and food insecurity (15). This sub-population, therefore, needs to be included when thinking about health policies and designing studies on health behavior.

Kenya is among the countries within SSA where a transition in physical activity and nutrition has been documented (16–18) and it is the country of focus in this study. Adolescents in Kenya bear the brunt of this transition as national survey data show that 61% of adolescent girls and 36% of the adolescent boys (15–19 years) do not optimally engage in continuous physical (19). Prevalence of overweight was also documented at 11% among the adolescent girls (19), and other studies indicate an increasing consumption of processed foods in Kenya (20). Disproportionately higher prevalence of physical inactivity and overweight has been documented at the Kenyan coast. For instance, a prevalence of 71.9% of physical inactivity among adult women (15–49 years) at the Kenyan coast was much higher than that in the Western (39.1%) and Nyanza (51.4%) regions of Kenya (19). This stated, research on the topic of diet and physical activity within the Kenyan context has not focused explicitly on adolescents, despite systematic investigation of the underlying factors for this burden (16–20). Furthermore, research focused on diet and physical activity behavior of Kenyan coast dwellers remains limited. In such coastal settings where the majority of the population resides in peri-urban and rural settings characterized by extreme poverty (17), there is an urgent need for a systematic investigation of which factors and how they influence young people’s diet and physical activity. Specifically, published research is almost non-existent among adolescents at the Kenyan coast and yet this developmental period is a window of opportunity during which interventions can readily contribute to breaking a vicious cycle of poor dietary behavior, sedentary lifestyle, poverty, and chronic disease (2, 5, 9).

To improve understanding of the underlying factors for poor dietary and physical activity in adolescents, and to guide the design of suitable interventions to tackle such behaviors, it is crucial to utilize a robust approach such as the ecological model (21). To address this gap, we first conducted a qualitative study with an ecological approach to understand young people’s and stakeholders’ perspectives on unhealthy diet and sedentary lifestyle of adolescents (10–19 years) in Kilifi County at the Kenyan coast. To inform future work, which will involve health risk behavior tool adaptation and validation for adolescents in Kilifi, this study’s participants comprised of a diverse group of young people, such as school going adolescents, adolescents living with HIV, and adolescents who had dropped out of school from Kilifi County. Specifically, we explored perceptions regarding: (i) adolescents’ unhealthy food choices and their forms of sedentary behavior; (ii) predisposing factors; and (iii) protective factors against unhealthy food choices and sedentary behavior among adolescents. We explore the variety and the convergence of the participants’ perspectives and the factors underlying unhealthy diet and sedentary lifestyle of adolescents within this setting.

### Theoretical Framework

This study draws on the Social Ecological model by McLeroy and colleagues (22) to examine the factors underlying unhealthy diet and physical inactivity of adolescents in Kilifi. The model borrows from earlier work of Bronfenbrenner (21), which explains that human development is influenced by a multi-person system of interaction and several aspects of the environment beyond the immediate situation of the individual. The Social Ecological model hypothesizes that there are five levels that interact to determine human behavior: intrapersonal factors that comprise biological and personal history factors, such as attitudes, income, education level, and knowledge; interpersonal factors that include support systems, such as family, and friendships, and formal or informal social networks; institutional factors that include social institutions with organizational characteristics, formal or informal rules and regulations; community factors that involve relationships among informal networks, organization, and institutions; and public policy factors that include local, state, and national laws or policies. For the purpose of our study, we merge the institutional and community factors into a single level that we refer to as community or institutional factors. The use of the Social Ecological model has gained popularity and it has been used in various public health studies for instance on chronic diseases (23, 24) and substance use behavior (25, 26).

## Materials and Methods

### Participants

This study encompassed of two groups of participants: 78 adolescents and informants, consisting of young adult community representatives and 10 adults working with adolescents in Kilifi county, for example, employees of community-based organizations (*n* = 4), teachers (*n* = 3), county government staff (*n* = 1), and clinicians (*n* = 2). More details of the participants are provided in the results section (see Table 1).

**Table 1:** A stratification of the study sample by age, sex, and education level.

Participants	Age range (median age)	*N*(sex)	Education level
**Primary school adolescents**			
Peri-urban male students	10–14 (14)	7 (males)	Class 5–8
Peri-urban female students	10–13 (12)	7 (females)	Class 5–8
Rural male students	12–16 (13.5)	8 (males)	Class 5–8
Rural female students	13–16 (14.5)	8 (females)	Class 5–8

**Secondary school adolescents**			
Peri-urban male students	16–19 (17)	8 (males)	Form 1–2
Peri-urban female students	15–17 (16)	6 (females)	Form 1–2
Rural male students	16–18 (17)	9 (males)	Form 1–2
Rural female students	15–18 (16)	9 (females)	Form 1–2
Adolescents living with HIV	12–19 (13.5)	9 (5 males, 4 females)	Class 3–Form 1
School dropout adolescents	12–18 (14)	7 (5 males, 2 females)	Class 2–Form 2
Stakeholder Key Informants	27–51 (31)	10 (4 males, 6 females)	University degree (5), Post-secondary level (5)
Young adult community representatives (informants)	22–28 (25)	7 (3 males, 4 females)	College level (4), Secondary level (2), Primary level (1)

### Procedures

This specific study was part of a larger qualitative study conducted between August and November 2016 at KEMRI-Wellcome Trust Research Programme (KWTRP). The study was conducted in Kilifi County at the Kenyan coast, specifically within the Kilifi Health and Demographic Surveillance System (KHDSS) (27). A detailed description of the recruitment of study participants and data collection procedures for this qualitative study has been documented elsewhere (28). In summary, a snowballing process was used to recruit the study participants. A small number of researchers at the KWTRP in Kilifi whose work involved adolescents were initially contacted and they recommended additional key informants with experience working with adolescents in the similar setting. This process was repeated with all new interviewees so as to identify more participants sharing relevant perspectives for the study. In the schools, head teachers suggested a teacher who served as a key informant. Young adults were purposively selected as informants using an existing data base of around 200 community representatives in the Community Engagement department at KWTRP (29), taking into account their residential area (peri-urban or rural settings), education status, biological sex, and religion so as to reflect the diversity of the population.

School going adolescents were purposively identified from two primary and two secondary schools in the KHDSS area. These schools were chosen through consultation with the Kilifi County Education Office and researchers at the KWTRP with ongoing work within the school setting. Specifically, these schools were located in either a peri-urban or rural setting, and these were day schools attended by both male and female students. Kilifi town center was the reference peri-urban setting and areas within 10 or more kilometers and having limited social services were considered as rural settings. Upper-primary school students (i.e., from Class 5 to 8) and lower-secondary school students (i.e., from Form 1 and 2) were purposively recruited from class records with a specific consideration of sex, religion, and age.

Adolescents who dropped out of school were recruited with assistance from the County department of Public Health’s community support staff. Recruitment of the school dropout adolescents was done by purposively selecting them from two community health catchment units of Kilifi with consideration of age, sex, and religious diversity.

The recruitment of adolescents living with HIV was done through the youth club at the HIV care clinic at Kilifi County Hospital and some were recruited directly from the community through home visits to families of HIV-infected adolescents by a worker from the clinic.

All adolescents, their caretakers, young adults, and key informants that took part in this study were appropriately informed about what the study entails, potential risks, and benefits of their participation prior to obtaining their written informed consent.

We conducted focus group discussions (FGDs) lasting about 75–120 min with the adolescents and young adult community representatives. Each key informant was also administered to a 60- to 90-min-long in-depth interview at their time of convenience in a preferred venue.

The FGD guide and qualitative interview guide were both developed utilizing materials on health risk behavior from the World Health Organization and the Centers for Disease Control and Prevention documents (30, 31). We utilized an open-ended question followed by further probing that asked participants to explain specific forms of risk behavior which they perceive as commonly undertaken by adolescents aged 10–19 years who live in Kilifi. The views documented in this manuscript were spontaneous. Permission was asked from all participants to take notes and to audio record the discussions and interviews conducted.

### Data Analysis

Verbatim transcriptions of the audio-recorded interviews and discussions were conducted by a professional team which were later translated into English. The translated scripts were scrutinized by two authors (Derrick Ssewanyana and Patrick N. Mwangala) who also developed initial codes from *a priori* and emergent issues. Using NVivo 11 software (QSR International Ltd., Southport, UK), final coding was conducted by Derrick Ssewanyana. This was later followed by a series of discussions by the research team about the codes and consensus was reached on developing them into themes with guidance from the Socio Ecological model (22). A case- and theme-based approach was used to chart the data using Microsoft Excel. This framework was later reviewed, discussed, and agreed upon by the research team and used as a basis for interpretation of the results. A descriptive analysis summarized participants’ demographic characteristics and the perceptions identified during FGDs or key informant interviews.

## Results

A stratification of the study sample by age, biological sex, and education level is summarized (see Table 1). In total, 78 young people took part in 10 FGDs (eight of these were sex disaggregated). Also one FGD was done with seven young adults and 10 in-depth key interviews were held with three teachers in charge of student guidance and counseling; four employees of community-based organizations; one County government staff; and two County hospital staff.

### Forms of Unhealthy Diet Prevalent among Adolescents in Kilifi

Overall, there was general agreement that certain foods are unhealthy, though favorite among adolescents at school or in the community. For example, in 72% of the FGDs and 90% of the key informant interviews, deep fried potatoes such as street prepared “viazi karai” and potato chips sold in restaurants and hotels were discussed as commonly consumed by adolescents despite being unhealthy foods. Likewise, in 55 and 70% of the FGDs and key informant interviews, respectively, commercially processed juices and sugar dense confectionaries, such as cakes, sweets, and ice-cream, were discussed:

Here my boys and girls in class 8, you find that instead of them buying ‘ugali’ (staple food made from maize flour) and beans, you find them buying a bottle of juice made from that chemical (referring to a certain brand of processed juice) and deep fried potatoes (KI, primary school teacher, male, 48 years).

Furthermore, three key informants who were secondary school teachers, and young people from 45% of the FGDs (mainly male primary school students, young adults, HIV-infected adolescents, and adolescents who had dropped out of school) pointed out that even when it comes to typically traditional meals, there is a common tendency for adolescents to consume an unbalanced diet, especially dense in starch or carbohydrates although this dietary practice was mainly associated with poorer households and rural dwellers:

For food, you ideally are supposed to be changing it now and then, but you may find the food is expensive, then you’ll be forced to eat ‘sima’ (staple food made from maize flour) daily and not any other food (Group 10, adolescent who dropped out of school).

### Forms of Sedentary Behavior Prevalent among Adolescents in Kilifi

**Figure 1:**
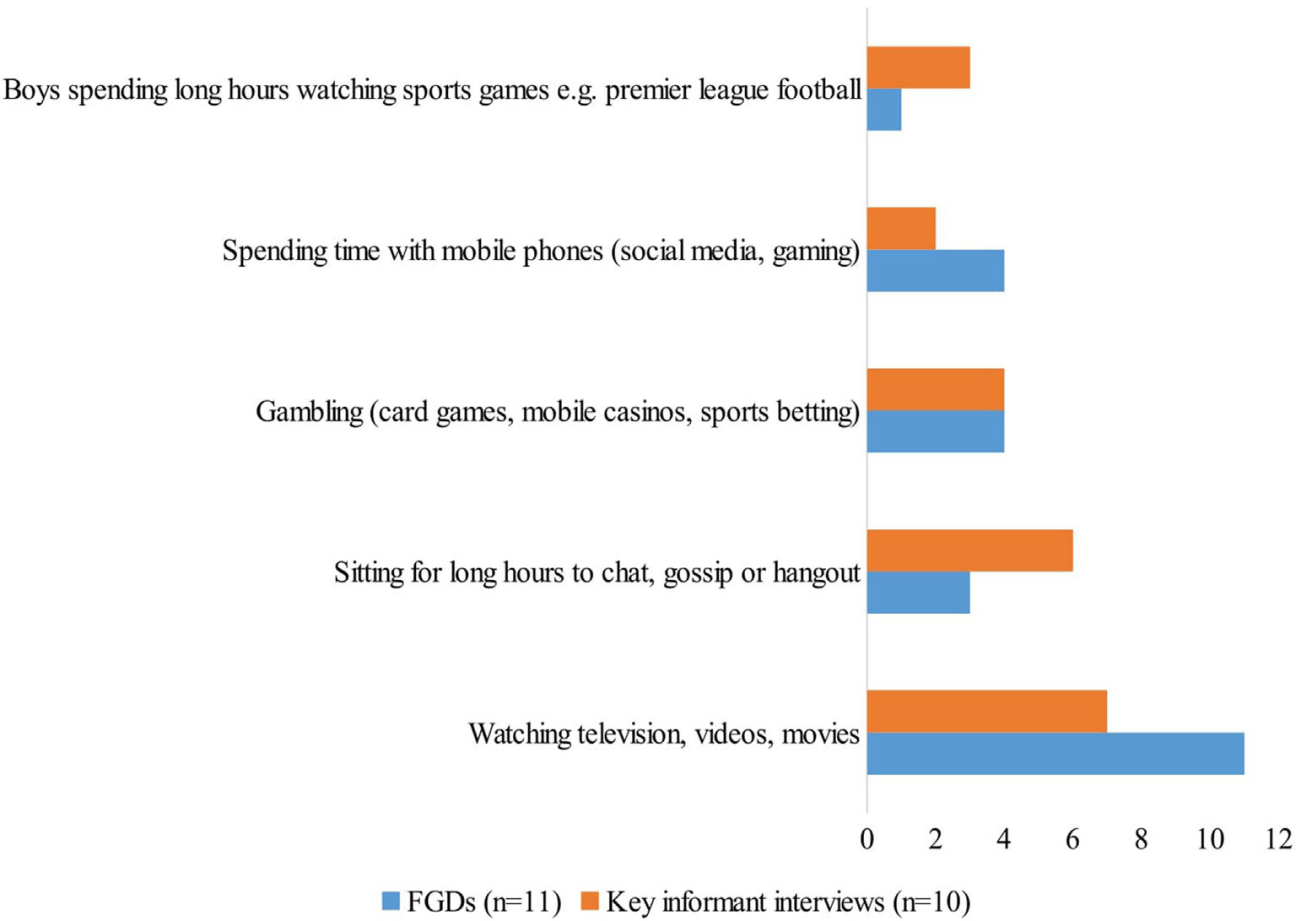
A summary of perceived prevalent forms of sedentary behavior in adolescents in Kilifi.

Various forms of perceived sedentary behaviors were identified by the participants and these have been summarized by number of FGDs and in-depth key interviews during which these forms of behavior were discussed (see Figure 1). One noteworthy behavior is gambling, which was discussed among 36% of the FGDs especially young adults and rural primary and secondary students, and by 40% of the key informants as an emerging form of sedentary activity that takes up adolescents’ time for physical activity, especially in the town centers. Among the common gambling activities mentioned were card games (locally referred to as “kamare”), sports betting and a form of casino which they described as portable small gambling machines increasingly common in the town centers:

I realized it was a casino, I could count 30 boys and am sure even if you drive there now, you will find them there. They put 20 shillings then they play the casino (KI, Clinician, female, 30 years).

### Underlying Factors for Unhealthy Diet among Adolescents in Kilifi

**Figure 2:**
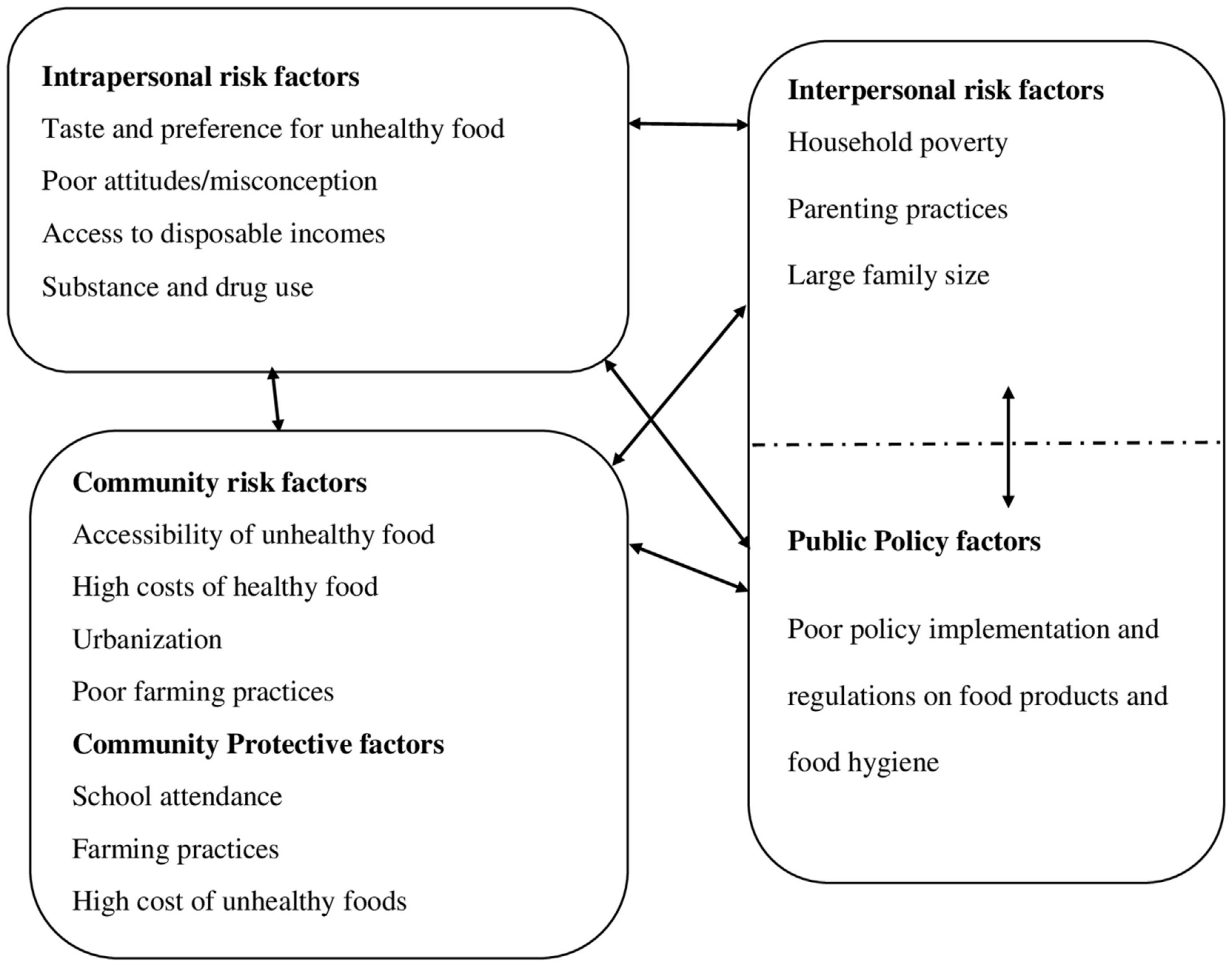
Factors suggested to underlie unhealthy diet among adolescents in Kilifi.

Respondents’ views surrounding both risk and protective factors for an unhealthy diet of adolescents are categorized into intrapersonal, interpersonal, community, or institutional, and public policy domains of the Socio Ecological model (22). Overall, a comparable number of risk factors were discussed at the intrapersonal, interpersonal, and community levels, with fewer issues raised regarding the public policy domain. With the exception of the factors at the community level, there was hardly any mention of protective factors at other levels (see Figure 2).

### Intrapersonal Factors Regarding Unhealthy Diet

#### Individual Preferences

Participants from 5 of 11 FGDs (primary and secondary school students, adolescents living with HIV, adolescents who dropped out of school, young adults) and three key informants indicated that the taste of certain foods, as well as their method of preparation are important factors that compel adolescents toward making unhealthy food choices. They discussed that fatty foods and processed products have an appealing taste, as compared to traditionally prepared foods:

Nowadays people don’t like taking the traditional foods, they despise the ‘mboga’ (green vegetables), they say it’s not sweet. So they prefer taking the junk food like pizzas. They take junk food over the traditional food (Group 6, female, peri-urban primary school).

#### Attitudes and Misconceptions

Particular negative attitudes were thought to discourage adolescents from consuming healthier traditional food stuff. Among those mentioned were the tendency to associate consumption of traditional foods with being a person of low social status:

It gives them a feeling that if you are a modern person of this era you have to take things like ice-creams and the sugary foods not knowing that its almost opposite with your health (KI, County government staff, male, 30 years).

#### Disposable Incomes

Two key informants (a secondary school teacher and a CBO employee) and several primary and secondary school students from three FGDs pointed out that when adolescents access money either as direct handouts from parents for upkeep or through casual labor, they can readily access the earlier discussed unhealthy foods.

Some parents are working for long, from morning to evening and during lunch time, they will give their child money for food. He will go and buy junk food because junk food is always ready (Group 5, male, peri-urban primary school).

#### Drug and Substance Use

Some peri-urban primary and secondary students discussed that the loss of appetite resulting from use of addictive substances such as khat and cigarets contributes to poor dietary behavior:

There are some drugs like khat which when used, you will lose the appetite of eating. So when somebody uses them and then goes to the hotel, he may even just eat one ‘chapatti’ (a common form of bread beaked from flour) (Group 8, female, peri-urban secondary school).

It can be noted that some factors in this domain may co-occur or be casually related. For instance, when adolescents have disposable income, they are likely to be able to afford certain unhealthy diets and may use substances which may negatively impact on their dietary behavior.

### Interpersonal Factors Regarding Unhealthy Diet

#### Household Socioeconomic Status

In 46% of the FGDs (rural primary and secondary students, adolescents living with HIV, adolescents who dropped out of school and young adults) and in 30% of the key informant interviews, the lack of household income to purchase nutritious food or ensure food security in the home was discussed as a major risk factor for poor dietary behavior, such as low fruit and vegetable intake, and the consumption of an unbalanced diet dense in carbohydrate:

The good foods that one is supposed to take, they do not if their family cannot afford, so one eats what they can afford (Group 3, male, rural secondary school).

On the contrary, two peri-urban secondary school adolescent girls expressed that wealthy households are more likely to consume unhealthy diets such as sweetened confectionaries, such as cakes and ice-cream. Thus, they urged that better household socio-economic status could as well predispose adolescents to unhealthy dietary behavior.

#### Parenting Practices

Views from a medical social worker, female secondary school teacher, and a few male students from a peri-urban school around parenting pointed to a number of predisposing factors, such as the lack of strict parental monitoring of adolescents’ dietary habits; parents’ tendency to spend less time together with their children; some family heads wasting their finances on unnecessary expenses and, thus, failing to provide food in the household; and the practice of some parents providing money to their children instead of providing them prepared foods.

It is important to note the interrelatedness of these factors within this domain, for example, household poverty, as well as intrapersonal factors such as adolescents’ access to disposable incomes. Parenting practices may as well play a major role in shaping certain attitudes and norms surrounding dietary choices.

#### Having a Large Family Size

To a lesser extent (i.e., by two key informants), having a large family size was discussed as one of the risk factors for poor dietary behavior. They pointed out that it is common to find large sized extended families in Kilifi, and that this creates challenges for optimally addressing the nutritional needs of family members.

### Community or Institutional Factors Regarding Unhealthy Diet

#### Food Accessibility

Discussions regarding factors related to the community domain were dominated by considerations of various aspects of accessibility. Adolescents in six of the 11 FGDs (peri-urban primary schools, rural and peri-urban secondary schools, and adolescents who dropped out of school) and three key informants indicated that there is a wide variety of unhealthy food products, which can be easily accessed in the Kilifi community. They also highlighted that unhealthy foods are increasingly affordable, even for people of lower socio-economic status. Peri-urban primary students also stressed that the cost of certain healthy foods is high which discourages their consumption:

I think sodas are doing well and that’s keeping people off from taking healthy juices and I think also they are affordable compared to a glass of juice. And junk foods or fast foods also, they are available and they are cheap (KI, Clinician, female, 30 years).

Several rural primary school male students and one male peri-urban primary school teacher expressed divergent views that in Kilifi nutritious and traditional foods, such as vegetables, fruits, and cereals, are readily accessible, especially in the rural settings. In addition, a key informant who headed a CBO reported that many unhealthy food products such as fast foods are expensive and unaffordable to many adolescents in Kilifi.

#### Farming Practices

Both risk and protective factors related to farming practices were discussed. Two key informants who were employees of CBOs expressed that that poor farming practices characterized by the lack of vegetable and fruit gardening in many homes in Kilifi which limits their accessibility to nutritious diets.

A rural primary and secondary school participant and two key informants had divergent views and pointed out that keeping livestock and poultry, and subsistence farming are common in Kilifi and avail healthy food products for many households. They further emphasized that, during a good harvesting season, fruits and other foods is in plenty supply and readily accessible by adolescents in Kilifi.

#### Urbanization

Urbanization of Kilifi was noted as an important risk factor by one key informant who was a peri-urban primary school teacher. He explained that urbanization comes with increased supply and demand of unhealthy foods and also an increase in infrastructure, such as super markets, kiosks, and restaurants where such food products are accessible.

#### School Attendance

School attendance was cited as a protective factor by one key informant who was a secondary school teacher. He reported that schools are important in enabling healthy diets; through educating adolescents on proper dietary behavior, as well as ensuring that school meals provided to students are well balanced.

### Public Policy Factors Regarding Unhealthy Diet

One key informant, who was a peri-urban secondary school teacher, highlighted that policy and regulations regarding food products and food hygiene were poorly enforced, which results in rampant marketing of unhealthy and unhygienic food product in the community:

The idea of food here is not regulated, even the hygiene. Anybody can sell anything at any point and the person is not answerable. In fact that is one of the major failure we have here. There are no designated of food eateries (KI, secondary school teacher, female, 51 years).

#### Factors Regarding Sedentary Lifestyle among Adolescents in Kilifi

**Figure 3:**
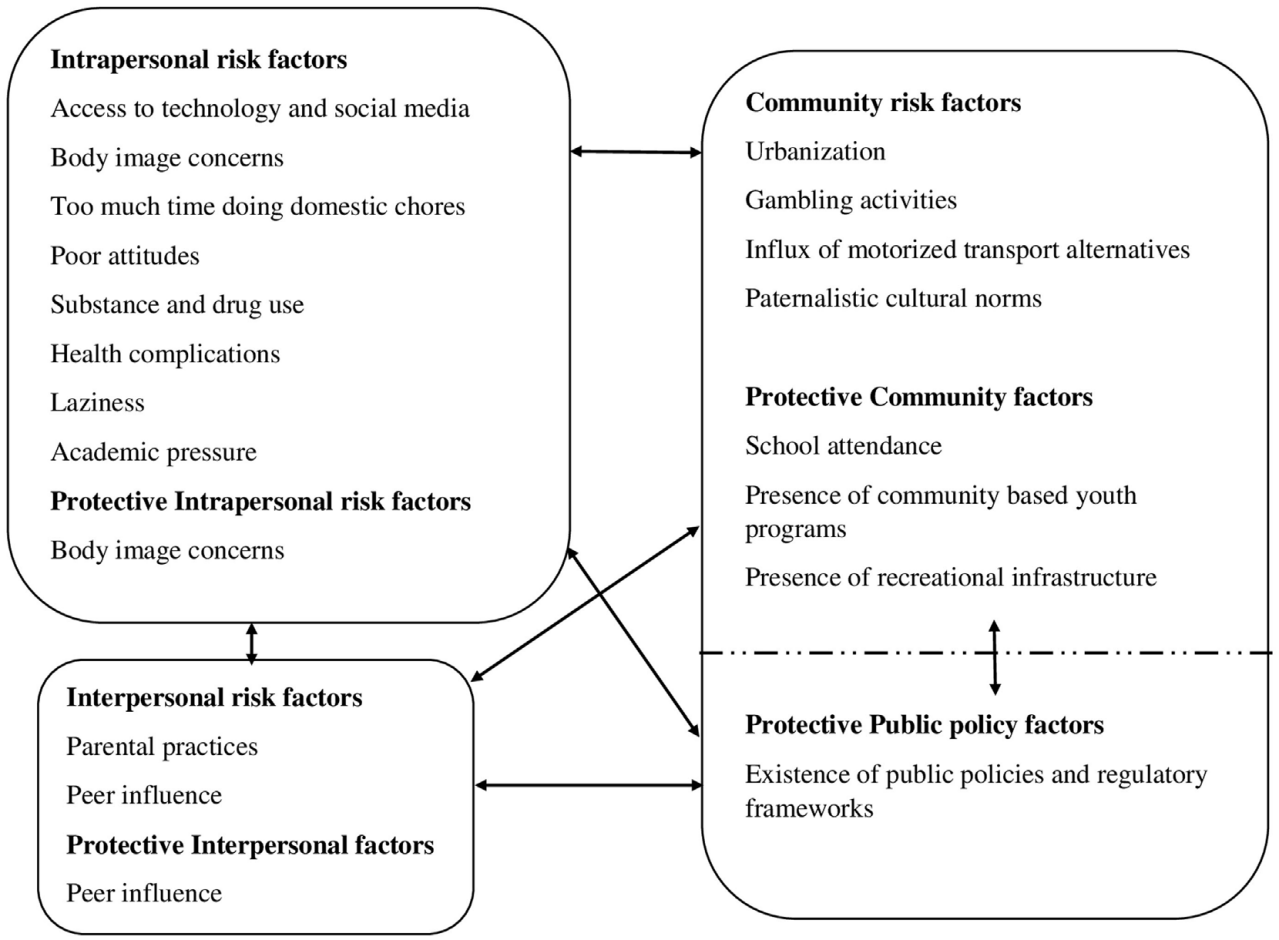
Factors suggested to underlie sedentary lifestyle among adolescents in Kilifi.

Discussions regarding perceived underlying factors for sedentary lifestyle were mainly dominated by intrapersonal and community level factors. Minimal factors were mentioned at the interpersonal and public policy level (see Figure 3).

### Intrapersonal Factors Regarding Sedentary Lifestyle

#### Domestic Chores

Although there were some views that some domestic chores are forms of physical activity, some adolescents who comprised rural secondary school students, adolescents who had dropped out of school and adolescents living with HIV, as well as two key informants pointed out that these activities may result in some level of physical inactivity in some adolescents, especially girls to some level of physical inactivity. They said that some of this work is strenuous, is too much and consequently diminishes young people’s time and opportunity for exercising:

Most house chores involve washing clothes so if the clothes are many you get busy washing them leaving no time for exercise (Group 3, male, rural secondary school).

#### Increased Access to Technology and Social Media

Increased access to technology and social media by young people in Kilifi was discussed in 40% of the key informant interviews and in 3 FGDs comprising rural secondary, peri-urban primary, and peri-urban secondary school students as an important risk factor for a sedentary lifestyle. Through personal access to gadgets such as mobile phones, increased presence of entertainment and internet service providers in the area, and access to devices such as televisions at home, adolescents were presumed to spend long sedentary hours using such technology and media:

So many people chat to an extent that they end up forgetting about exercising. That is because they are always busy with their phones chatting (Group 4, female, rural secondary school).

#### Body Image Concerns

Body image concerns such as shyness to expose certain body parts during games and physical activities especially by adolescent girls were raised during discussions with peri-urban primary and secondary school students and adolescents living with HIV. They also expressed that the feeling of embarrassment about some adolescents’ body weight (for example, those who are overweight) would hinder their engagement in physical activity.

However, there were some divergent views from a few peri-urban primary and secondary school students who said that some of the adolescents actually exercise because they are not comfortable with their body image and would want to become physically fit (i.e., build muscles, or reduce body weight) and more attractive.

#### Drug and Substance Use

Drug and substance use of alcohol, marijuana, cocaine, and tobacco by adolescents was discussed in five out of 11 FGDs with young people and by three key informants as a predisposing factor for a sedentary lifestyle. They explained that substance use leads to weakness, dizziness, and sleepiness, resulting in physical inactivity:

Especially once you take a puff of bhang, you just feel dizziness and you just sleep and forget about doing physical exercises (Group 1, male, rural primary school).

#### Health Complications

Health complications, such as asthma, underweight, and using medication, were some factors cited by adolescents living with HIV, and rural and peri-urban primary school students, who felt that such conditions could hinder some of adolescents from taking part in physical activities:

Some use drugs (medicines) which they take due to their health status don’t allow them to do such exercises (Group 5, male, peri-urban primary school).

There are other who, I mean, their health does not allow them. For example if you have a small body and then you go to do gym, won’t you just fall there? (Group 9, adolescent living with HIV).

Other risk factors for physical inactivity discussed by several study participants included laziness of some adolescents; academic pressure that leads to less or no time for physical activity; being distracted by romantic relationships; and poor attitudes or misconceptions, for instance, that physical activity is a waste of time and may lead to dirtiness.

### Interpersonal Factors Regarding Sedentary Lifestyle

#### Parental Practices that Condone Physical Inactivity

Parental practices that condone physical inactivity were pointed out during five FGDs with rural and primary school students, adolescents living with HIV and adolescents who had dropped out of school, and also in two key informant interviews. These parenting practices included punishment of children whenever they go for sports; and confining children within the home and refusing to grant them permission to go for sports activities.

#### Peer Influence

Peer influence was discussed as both a risk and protective factor by a clinician and a secondary school teacher, and by adolescents that had dropped out of school. It was pointed out that adolescents sit and hangout or gossip in small groups for long hours without performing any form of physical activity. However, it was also discussed that adolescents, especially males, tend to join sports teams or other activities, such as weight lifting, which keep them physically active.

### Community or Institutional Factors Regarding Sedentary Lifestyle

#### Gambling

Gambling was discussed in 36% of the FGDs and by 40% of the key informants as an increasingly growing problem in Kilifi, characterized by the rapid establishment of affordable gambling options that are poorly regulated, appealing to young people and take up their productive time:

The Asians have brought these casinos and they have put up two or three or whatever number at every bus stop. These ones have greatly contributed to some people not working; they go there at 6am and leave there at 6 pm (Group 11, female, community representative).

#### A Paternalistic Culture in the Community

A paternalistic culture in the community was described, by three female key informants and some rural primary school female students, as treating boys differently from girls, whereby boys are not obliged to actively participate in domestic chores (some of which were considered as a form of physical activity). It was also pointed out that boys are less monitored compared to girls and, thus, may find more time to engage in sedentary behavior, such as watching movies and soccer matches in video cafes.

So you will find that if a girl goes to the movies, when she gets back home she is questioned but when a boy goes he spends all the time and he is not questioned (Group 2, female, rural primary school).

A boy child is raised minus home duties, it is like these activities are for females. They can’t wash utensils they can’t clean the house. Indeed, most of the times I think male child are not engaged in domestic chores (KI, Clinician, female, 30 years).

#### Influx of Motorized Transportation Alternatives

Influx of motorized transportation alternatives such as motorcycle taxis (locally known as boda-boda or tuk-tuk), and mini vans was raised by peri-urban primary school students and one secondary school teacher as a factor that interferes with active transportation of many adolescents in Kilifi and thereby contributing to reduced physical activity:

One of the major physical activity that we have here is walking but there is an influx of boda-boda (motorcycle taxi). That is one thing that limits their physical activity (KI, secondary school teacher, female, 51 years).

In the FGDs and key informant interviews, some protective factors at the community level were discussed.

#### School Attendance

School attendance was recognized by rural and peri-urban primary school students and by three key informants as a protective factor that offers various opportunities for learning and engaging in physical activity. For instance, these participants pointed out that most schools in Kilifi have physical education (P.E.) in their curricula, students have some sports’ facilities such as playgrounds, and schools host or participate in local and national sports competitions. These factors were thought to promote adolescents’ physical activity. However, there were several peri-urban primary school students who reported that at times schedules meant for physical activity are not observed since certain teachers use that time for extra teaching hours:

Some teachers will always take the free time of the children, like physical education (P.E), lunch time, break time and they will come to the class to teach because they want their subject to be the top (Group 5, male, peri-urban primary school).

#### Presence of Community-Based Youth Services and Communal Infrastructure

The presence of some youth sports clubs, communal infrastructure such as football pitches, and community-based or non-governmental organizations (CBOs or NGOs) which provided sports equipment and various social services was discussed by 50% of the key informants as an important protective factor against physical inactivity in Kilifi:

But we have another organization which is called Move the Goal Posts. Have you heard about that? Now this one has really trained girls in football (KI, primary school teacher, male, 48 years).

### Public Policy Factors Regarding Sedentary Lifestyle

#### Existence of Government Policy and Regulations on Access to Gambling Venues by Minors of Minors in Gambling

Only one key informant who was head of a community-based organization in Kilifi discussed a protective factor against physical inactivity at a public policy level:

But nowadays the government is very strict. We have a certain age of the boys and girls who are not supposed to be found in these video cafes especially during school time (KI, CBO director, female, 46 years).

## Discussion

Our results show a perceived preference of unhealthy, less traditional diet, such as fatty street foods, sweetened beverages, and sugar dense confectionaries, especially among the adolescents in urban settings. Our findings are aligned with reports of high consumption of sweetened beverages, junk foods, and inadequate fruit intake among school children (8–11 years) living in the Kenyan capital (32), and findings from a Kenyan household survey (20). Our findings underscore the increasingly documented nutritional transition in the SSA region (8, 33).

Extensive hours spent engaging in sedentary activities, especially the use of technology and socio-media, for example, television viewing, use of mobile phones, and watching sports, mainly by the young people in urban setting, was seen as a problem. Our findings concur with results from a study in Kenya that reports a high prevalence (13%) of television viewing for more than 11 h per week among urban children of 9–12 years (18), and passive leisure chatting on the phone, accessing web sites, and playing mobile-games. Such activities are found to negatively impact engagement in active leisure activities among Kenyan youths (34). Research emanating from other contexts has also emphasized the disruptive effect of technology use (i.e., mobile phones, television screening, and gaming) on physical activity, cardio respiratory fitness, and other health outcomes among youths (35–37). However, such research is still limited in developing countries such as Kenya.

Our results show that substance use as well as poor attitudes or misconceptions in relation to physical activity and consumption of traditional food are commonly perceived as intrapersonal risk factors for both unhealthy diet and physical inactivity among adolescents in Kilifi. Considering that there is a general increase in alcohol, tobacco, and illicit substance use among Kenyan youths, characterized by an early age of onset estimated at the age of 10 years (38), it is not surprising that negative impacts on diet and physical activity are commonly recognized in this setting. The findings also highlight the perceived co-occurrence of health risk behaviors and a potential for multi-component interventions to address such behaviors (39, 40). Our findings on adolescents’ poor attitudes surrounding diet and physical activity are consistent with findings from a study conducted among young adolescents in Nairobi, which found that 65% of participants had poor attitudes toward healthy diets, despite that their knowledge on nutrition was good (32).

At an interpersonal level, parenting behavior, such as providing money as opposed to prepared lunch food for their children, subjecting children to onerous domestic chores and placing unrealistic restrictions on adolescents’ time for physical activity, especially for the girls, stand out as commonly perceived, major risk factors for both an unhealthy diet and physical inactivity among adolescents in the Kilifi setting. Similar to our findings relating to parental influence, a 2016 Kenyan national report (41) considered the influence of family and peers on physical activity of children and adolescents (5–17 years) “poor.” The authors in the national report pointed out that many Kenyan parents lack adequate knowledge of their children’s level of physical activity and dieting, as well as of basic requirements for achieving better physical and nutritional health outcomes (41). Similar concerns have also been reported based on other Kenyan studies (32, 42), which underscores the need to target parents and caretakers with health promotion messages that emphasize the value of a healthy diet and sufficient physical activity of their children and other family members. Schools need to actively engage parents or caretakers in planning, supporting, and decision making on adolescents’ extracurricular activities and school-based approaches such as healthy food choices at school canteens, physical activity facilities, and designing sports’ curricula.

Food availability plays a key role in predicting dietary behavior of adolescents. Availability is principally determined by food prices, prices of alternative food products (i.e., processed foods), demand and supply, seasonality, and farming practices. Indeed, studies have found an adverse association between food insecurity and dietary patterns (43), and lower prices of healthy foods are more likely to predict healthier food choices (44). Again, this finding points to the relevance of multi-sectorial approaches (i.e., engaging agricultural, public health, and other sectors) in addressing dietary behavior of adolescents. Moreover, with poverty being a major concern in the study’s context, there is need to prioritize sustainable youth employment policies and programs.

Gambling was another issue highlighted in our findings, and one that is currently documented as a rapidly growing concern in Kenya and other parts of SSA (45). Studies of Kenyan adolescents are still scant but studies of young adults show a significant burden of gambling problems (46). Our findings are aligned with findings from research in Ethiopian school going adolescents of which almost 73% had ever gambled and 7% were pathological gamblers (47). In addition, common gambling options, such as card games, casino, and other traditional games, access to mobile phones, internet, and social media has made closed forms of gambling alternatives for many young people in SSA (48). A need for collaborative efforts across the private and government sectors as well as civil society in implementing feasible social policy and public health interventions to address gambling problems among young people is timely.

Similar perceptions relating to the underlying factors for diet and physical activity of adolescents were held by adolescents living with HIV and those not infected by the disease. We also did not find major differences relating to adolescents’ perceptions of underlying factors for sexual risk behavior in another study (28). Noteworthy, both groups recognize that disease-related conditions, such as long-term medication and low body weight, are risk factors for adolescents’ sedentary lifestyle. Other studies also note that chronic conditions such as HIV present a significant risk for suboptimal adolescent development characterized by psychosocial issues, risk taking behavior, and poor health outcomes (14, 15). There is, therefore, a need to consider adolescents with chronic conditions such as HIV when designing studies, programs, and policies to address health behavior.

Our findings emphasize the perceived protective role of school attendance for both dietary behavior and physical activity in adolescents and are aligned with those of a Kenyan report on physical activity of children and adolescents that ranked the school environment (i.e., infrastructure, programs, and policies) as “fair” (41). This was largely attributed to the Kenyan government policy that requires allocating at least 35 min for physical education classes (PE) at least three times a week (19, 41). Moreover, there is great public support for PE and the majority of Kenyan primary and high school students report having actively participated in such school programs (13, 18). School food programs have also been found to have a positive impact on school enrollment and nourishment in SSA (49). In Kenya, more than 1.5 million school children obtain a hot meal each day at school and there have also been efforts to integrate programs to ensure community food and nutrition security with school food programs, although sustainable financing of school food programs remains a major challenge (50).

### Limitations

Our study is limited by the reality that some of the views on dietary behavior and physical activity shared by our participants may be specific to adolescents living at the Kenyan coast in Kilifi. In other settings, processes may be different and, therefore, our findings cannot be generalized to other settings. Furthermore, since young people’s views were perceptions of their peers’ behavior, it is likely that some of their understandings were either an underestimation or overestimation of the situation of adolescents in this study’s context. However, it is important to note that we included a diverse mix of adolescent subgroups who were carefully selected to reflect diversity in the adolescent population of Kilifi and their opinions contrasted with those of key informants who were adults that worked extensively with adolescents in Kilifi County. In addition, we undertook a qualitative study and although we discuss our results in relation to other more quantitative studies, certain findings may need to be replicated in quantitative studies as well. Our findings identify the most important topics that may need to be addressed more specifically in future studies. The value of this in-depth process from a research perspective is that it enables researchers to generate knowledge, design quantitative studies and interventions that are more contextually informed and that focus on the specific challenges identified by the adolescents themselves and the members of the community.

## Conclusion

In our findings, a myriad of underlying risk factors was reported by adolescents and stakeholders, which include *intrapersonal* factors, such as substance use behavior, spending excessive amount of time on social media, poor attitudes, and preference for unhealthy diet; *interpersonal* factors, such as parenting practices such as subjecting children to onerous domestic chores and unrealistic restrictions especially on adolescent girls’ time for physical activity, and poverty; *community* factors, such as availability of unhealthy food options, gambling, and *public policy* factors, including poor implementation of policies and regulations regarding food products and food hygiene. Important opportunities, such as access to infrastructure and equipment for physical activity, education on healthy diet and physical activity, and a school food program, emanate from the supportive school environment and the existence of some community-based youth services. In order to counter unhealthy diet and physical inactivity among adolescents in Kilifi, we recommend targeting parents and caretakers with health promotion messages emphasizing the value of healthy diet and physical activity for their children; more active engagement of parents and caretakers in planning and supporting school programs on healthy diet and physical activity; and prioritizing sustainable youth employment policies and programs.

## Ethics Statement

All adolescents, their caretakers, young adults and key informants that took part in this study were appropriately informed about what the study entails, potential risks and benefits of their participation prior to obtaining their written informed consent. This consent was directly sought from participants aged 18 years and over but for those aged under 18 years, their parents or legal caretakers provided the consent. Adolescents’ assent was also sought from participants aged between 13 and 18 years. We obtained permission from the County director of education and head teachers at each school to involve schools in this study. The study was granted ethical clearance by the Kenya Medical Research Institute Scientific and Ethics Review Unit (KEMRI/SERU/CGMR-C/0047/3263).

## Author Contributions

This study was conceptualized by DS, AA, AvB, and CN. The study methodology was designed by DS and AA. DS and PM critically scrutinized the scripts and developed initial codes while DS did the final coding in NViVO, the data charting, and wrote the initial draft of this manuscript. Reviewing and editing of this manuscript was done by AA, AvB, and CN.

## Conflict of Interest Statement

The authors declare that the research was conducted in the absence of any commercial or financial relationships that could be construed as a potential conflict of interest. The handling Editor declared a shared affiliation, though no other collaboration, with three of the authors DS, AA and AVB.

## Availability of Supplementary Data

The supplementary data that support the findings of this study are available from the corresponding author upon reasonable request.
